# Effectiveness of hippotherapy on balance performance, neurophysiological parameters and clinical symptoms of multiple sclerosis: Study protocol of a randomized controlled multicenter study (MS-HIPPO II - Movement in Balance)

**DOI:** 10.1016/j.conctc.2025.101583

**Published:** 2025-12-05

**Authors:** Isabel Stolz, Leonard Braunsmann, Franca Rosiny, Dieter Poehlau, Thomas Abel, Volker Anneken, Marion Drache, Kristel Knaepen

**Affiliations:** a Institute of Movement and Neurosciences, German Sport University Cologne, 50933, Cologne, Germany; b Research Institute for Inclusion Through Physical Activity and Sports, Paul-R.-Kraemer Allee 100, 50226, Frechen, Germany; c DRK Kamillus Hospital, Hospitalstraße 6, 53567, Asbach, Germany; d Center for Therapeutic Riding Johannisberg e.V., Johannisberg 1, 53578, Windhagen, Germany

**Keywords:** Balance, Berg Balance Scale, Balance Perturbation, Brain Activity, EEG, Hippotherapy, Multiple Scerosis

## Abstract

**Background:**

Exercise and physical therapy have been shown to be effective in the non-pharmacological symptomatic treatment of multiple sclerosis (MS). Hippotherapy as an additive physical therapy intervention is applied in this study to promote balance and postural control, since the number of sufficiently high-quality randomized controlled trials indicating its effectiveness is limited.

**Objective:**

This study aims to provide more in-depth insights into the effectiveness of hippotherapy in MS in terms of balance and other patient-relevant outcomes, building on the results of a preliminary study (MS-HIPPO II, evidence level 1b).

**Methods:**

Based on a prospective, randomized, investigator-blinded, controlled multicenter study design, the primary endpoint of differences in balance will be investigated. Patients will be randomized to an intervention group (12 weeks of hippotherapy) or a control group (12 weeks of treatment as usual). Balance will be measured using the Berg Balance Scale (BBS) plus a standardized balance task on a force plate (AccuGait-Optimized, Advanced Mechanical Technology, Inc., Massachusetts, US) and a balance perturbation task on an oscillating sensorimotor therapy device (Bioswing Posturomed®, Haider, Pullenreuth, Germany). During balance tasks, electrocortical activity will be investigated using electroencephalography (EEG) (LiveAmp 32, Brain Products GmbH, Gilching, Germany). Secondary endpoints include fatigue (FSS), quality of life (MSQoL-54), pain (VAS), spasticity (NRS) and participation (WHODAS 2.0). Therapy progress will be documented via an ICF-based hippotherapy assessment-tool (EQUITEDO®, Frechen, Germany).

**Results and conclusions:**

The results should contribute to improve the understanding of non-pharmaceutical treatment options in the field of exercise and movement therapy in MS.

**Trial registration:**

The trial was registered at March 05, 2024 in the German Clinical Trial Register under DRKS00033449.

## 1 Introduction

For persons with multiple sclerosis (MS), perspectives on non-pharmacological symptomatic treatment have been increasingly investigated in recent years, in order to promote biopsychosocial functioning and maintain mobility [[1], [2], [3]]. To optimize health care beyond pharmacological therapy in outpatient treatment, MS represents one of 26 chronic conditions, where exercise is considered “medicine” [4]. In the development of the list of these 26 chronic conditions, cohesion for prescribing exercise as medicine was achieved on an evidence-based level [4,5]. It includes specific recommendations for type and dosage. Based on evidence of recent years, authors advocate exercise already at an early stage of MS, along with conventional medical treatment [6,7]. In this regard, physiologically oriented hippotherapy is practiced as a treatment for persons with MS, to improve movement processes by promoting the interaction of the central and peripheral nervous systems with the musculoskeletal system [8]. According to the German Federal Joint Committee (G-BA), hippotherapy is defined as physiotherapeutic treatment on a neurophysiological basis with and on a horse [9]. It is applied with the aim to counteract impaired or lost walking ability and to promote harmonious locomotion in a gait-physiologically correct movement pattern [8]. Specifically, the three-dimensional impulses from the horse's back in relation to centrifugal-, acceleration- and braking forces, e.g. by turns, gait changes or transitions, are expected to impact patients' body functions [8].

Prior studies have shown positive effects of hippotherapy in the complementary treatment of symptoms of MS, e.g. improving balance and postural control, reducing spasticity and self-perceived fatigue, increasing disease-related quality of life and function-related mobility [10,11]. In their systematic review focusing on motor functioning, Moutaftsis et al. [12] showed that hippotherapy had a positive impact on motor function, gait cycle (quality of walking), walking endurance performance, balance and orthostatic control. A further systematic review and meta-analysis by Suárez-Iglesias et al. [13] included 9 of 234 screened studies with sufficiently high methodological quality and confirmed previous evidence on improving static balance, fatigue and quality of life, but not the findings on gait quality and dynamic balance. In this context, the large number of studies not included in the reviews, indicates the widely diverging study quality of current investigations. The included studies had an average subject size of less than 30 patients (N = ⌀ 28.6), also different survey instruments were used and short therapy periods were evaluated, without follow-up assessments. The results of previous studies show, that despite the long-standing tradition of hippotherapy treatment in MS, there is still a lack of evidence of its efficacy based on randomized controlled trials (RCTs), study designs with sufficiently large sample sizes and studies with level 1 quality of evidence. The current study builds on the MS-HIPPO study (N = 70), which assessed the effectiveness of hippotherapy on the symptoms of MS, based on an RCT study design [11,14]. The two year study showed evidence of a positive effect of hippotherapy in MS patients at a high level of reliability (e.g. evidence level 1b) [11]. Central results showed that weekly hippotherapy, significantly improved balance performance (measured by BBS) as well as rapid fatigability (FSS), spasticity (NRS) and quality of life (MSQoL-54) in patients with MS [11].

## 2 Methods

The aim of the current MS-HIPPO II study is to quantify the effects of hippotherapy on balance control in MS patients by means of state-of-the-art biomechanical and neurophysiological measurements. The effects of hippotherapy will be assessed in a larger number of cases (N = 140), on evidence level 1b via an RCT study design, also including a follow-up measurement. The follow-up assessment is intended to provide information on the persistence of potential effects.

The outcome measures used in the first MS-HIPPO study will be extended in this study to biomechanical, as well as neurophysiological measurements, such as cortical activity, during balance and balance perturbation tasks [14]. The primary hypothesis to be tested is:

(1) Twelve units of hippotherapy significantly changes static balance performance, measured with the Berg Balance Scale (BBS) and a force plate, in the intervention group compared to the control group. Moreover, this change can also be sustained after the end of the intervention period (follow-up).

Secondary hypotheses are:

(2) Hippotherapy significantly changes the neuronal correlates of balance performance, measured with electroencephalography, in the intervention group compared to the control group.
(3) Hippotherapy leads to a change in MS specific symptoms such as spasticity, fatigue and pain in the intervention compared to the control group. Moreover, it is also expected that hippotherapy will positively influence motor functioning parameters, quality of life and participation in the intervention compared to the control group.

### 2.1 Design

**Fig. 1:**
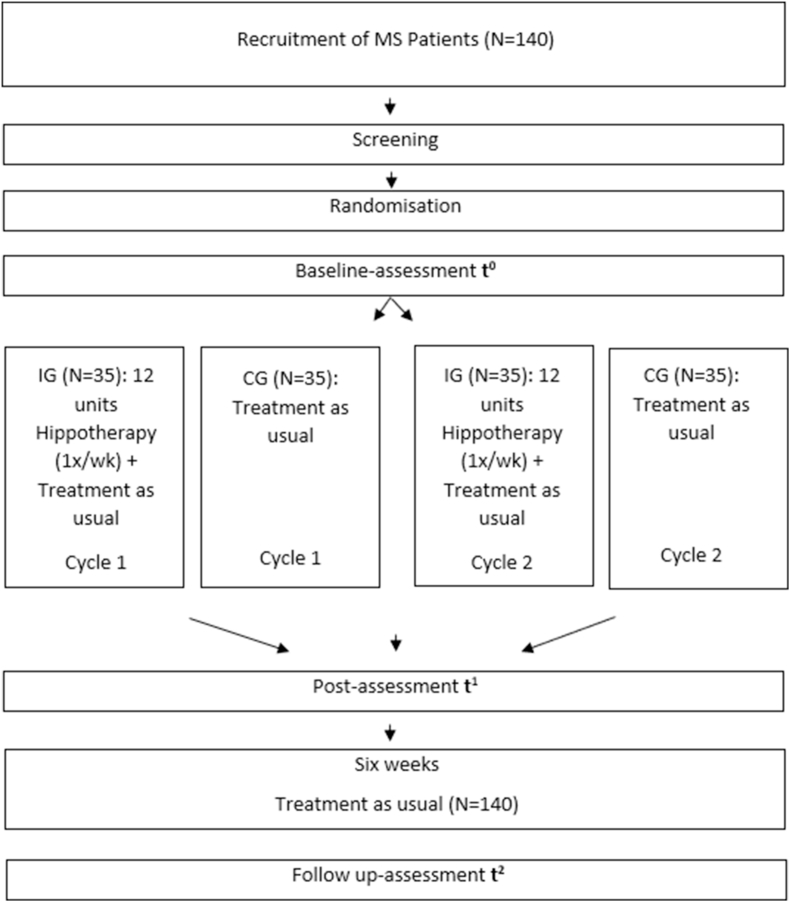
Study design.

A randomized controlled trial design will be applied to obtain data from in total 140 subjects: (1) hippotherapy intervention group (IG) (approx. N = 70) and (2) control group (CG) (approx. N = 70). Within a project duration of two years and six months (01.01. 2024–30.06. 2026), both groups, IG and CG, are to be assessed during two separate study cycles with IG (N = 35) and CG (N = 35) in each study cycle. Each study cycle will consist of a twelve-week intervention period (1x hippotherapy/week) with a pre/post measurement and a six-week follow-up measurement (see Fig. 1). Before therapy begins, the population will be randomly and evenly assigned to one of two groups: IG or CG.

### 2.2 Study sites

The study will be conducted at 8 study sites in Germany (see Table 1). Hippotherapy is carried out according to the methodology of the German Curatorship of Therapeutic Riding e.V. (DKThR), which ensures quality of therapeutic measures, animal welfare and standardization [15]. All facilities fulfill the requirements for study sites shown in Table 2 and are certified facilities for hippotherapy (DKThR)® by DKThR.

**Table 1:** Study sites.

Study sites
Center for Therapeutic Riding Johannisberg e.V., Windhagen
Center for Horseriding and Equine assisted Therapy, Carolinenhof, Essen
Center for Therapeutic Riding, Nuetzen
Equestrian Sports and Therapeutic Riding Center of Gold Kraemer Foundation, Frechen
HPZ Wolfgang Fahr Therapeutic Riding Ltd., Ostfildern
Riding Center Children's Aid, Ludwigshafen
Inclusive Equestrian Sports and Therapeutic Riding Center Karlshorst, Berlin
Gut Wittmoldt (estate), Wittmoldt

**Table 2:** Requirements for study sites.

Requirements study sites
DKThR-certified facility^a^ [16]
Implementation of hippotherapy according to the guidelines of the DKThR® [15]
Designation of the lead hippotherapist and a coordinator
Appointment of the responsible physiotherapist and his/her deputy
Separate room for baseline, post and follow-up assessments
At least 2 trained therapy horses must be available for the therapy
At least 6 patients must be treatable per week
Comprehensive cooperation with the monitor

a German Curatorship of Therapeutic Riding e.V. (DKThR) represents the national federation for equine-assisted therapy, rehabilitation and equestrian sports for persons with disabilities. The association comprises the fields of medicine, psychotherapy, psychology, education and sport [17].

### 2.3 Study population

Inclusion and exclusion criteria for participants are shown in Table 3, Table 4. Participants will be asked not to change or interrupt their existing physiotherapy or pharmacotherapy, e.g. their treatment as usual, during the data acquisition timeline. Written informed consent from participants will be obtained by a scientific study team.

**Table 3:** Requirement profile participant inclusion.

Inclusion criteria
Confirmed multiple sclerosis with spasticity of the lower limbs (all phenotypes)
Expanded Disability Status Scale (EDSS) score between 4 and 6
Written informed consent of the patient
Consent of the study site physician
Legal competency
Minimum age of 18 years

**Table 4:** Requirement profile participant exclusion.

Exclusion criteria
Hippotherapy within the last 12 months
Parallel participation in other intervention studies
Persons, who are in a dependent/employment relationship with the sponsor or investigator (potential conflict of interest)
Body weight over 90 kg
Acute exacerbation of the disease four weeks before the start of therapy
No balance while sitting
Planned start of treatment with new antispastic medication or with medication that may have an influence on test parameters (examples: Nabiximols (Sativex®), neuraxial administration of baclofen; 4-aminopyridine)
Seizure disorder not controlled by medication
No or insufficient ability to spread the legs so that the patient can sit on the horse
Known severe osteoporosis
Known severe osteoarthritis of the hip
Known severe scoliosis that could be aggravated by hippotherapy
Severe blood clotting disorders where there is a risk of hematoma due to hippotherapy
Insufficiently stable concomitant diseases in the field of internal medicine, gynecology and/or surgery
Allergy to horse hair
Pregnancy
No balance impairment (Berg Balance Scale over 50 points before the start of the intervention)

### 2.4 Intervention

Hippotherapy will be carried out once a week for 30 min additionally to patients‘ usual physio- and/or pharmacotherapy (1-2x/week). The vibrations of the horse's back and the rhythm of the horse's stride are being utilized to achieve physiological reactions to improve individual balance performance, mobility, muscle strength and an upright sitting position. It will be carried out as a complementary movement-therapeutic approach for study-related purposes and not part of the treatment routine of patients with MS in standard outpatient rehabilitation. Therapies are standardized according to the principles of DKThR and carried out by trained physiotherapists plus state-approved hippotherapy (DKThR)® license [8]. The Intervention manual for hippotherapy (DKThR)® can be found in Appendix A1. The CG will undergo their treatment as usual without additional hippotherapy. Treatment as usual is defined as the continuation of physiotherapy (1-2x/week) and/or pharmacotherapy. The physiotherapeutic treatment aims to improve individual mobility, build up reduced muscle strength, stretch shortened muscles and loosen tense muscles, as well as alleviate pain through massages and/or heat/cold/electrotherapy. This is supplemented by gait and mobility training (everyday mobility) as well as weight-bearing training, improved coordination and balance and stabilization training. The CG will be offered 5 hippotherapy sessions (30-min units once a week) as an incentive after completing the study and to ensure compliance with regard to ethical aspects. Changing inclusion and exclusion parameters will lead to a drop out. A change in medication or participation in another measure (e.g. study, rehabilitation activity) is not permitted during the trial. Participation in less than 9 out of 12 hippotherapy sessions will lead to a drop out. Pregnancy in participants will also lead to a drop out.

### 2.5 Outcomes

The primary endpoint of the study is the change in balance performance after 12 units of hippotherapy (change from baseline). To assess the primary outcome measure, the Berg Balance Scale (BBS) will be used in an independent assessment by a blinded observer (Table 5). Blinded assessors will be trained for collecting the BBS data prior to the start of the intervention by the investigators. Unblinding is not permitted during the trial. Assessors will be trained to collect EEG, force plate and Posturomed® data. Therapists at study centers will be educated and instructed on how to complete Adverse Event Report forms (AEs) and EQUITEDO® assessments.

**Table 5:** Protocol Berg Balance Scale.

(0–4 points/sum score)
1. from sitting to standing
2. standing without support
3. sitting without support
4. from standing to sitting
5. transfers
6. standing with eyes closed
7. standing with feet close together (close foot stance)
8. reaching/longing forward with outstretched arm
9. picking up an object from the floor
10. turn around to look backwards
11. turn 360°
12. alternately place your feet on a footrest
13. standing with one foot in front of the other (tandem stand)
14. standing on one leg (one-legged stand)

As the gold standard for measuring balance in a clinical setting, the BBS enables the results of this therapy to be compared with other interventions, and is therefore applied as primary outcome measure [18,19]. In regard to validity and reliability, the BBS shows satisfactory values [[18], [19]].

Additional to BBS, a balance task and a balance perturbation task will be implemented. The balance task will be performed on a force plate (AccuGait-Optimized (ACG-O), Advanced Mechanical Technology, Inc., Massachusetts, US). The task consists of: standing with eyes open and closed, closed stance, (semi-) tandem stance and (semi-) one-legged stance (Table 6). As the participant sways while standing on the force plate, the center of pressure (CoP) excursion in millimeters in all directions of sway (anterior–posterior and medial–lateral) will be measured with regard to the attainment of postural stability. In addition, brain activity will be recorded by means of electroencephalography (EEG) using a portable system with 32 electrodes (LiveAmp, Brain Products GmbH, Gilching, Germany). By measuring the excitatory postsynaptic action potentials, a movement-induced change in brain activity in the theta, alpha-1 and alpha-2 frequency bands is expected.

**Table 6:** Balance and balance perturbation task.

Protocol balance tasks (sequence of tests 5–7 randomized)
1. sitting - eyes open - 120s
2. sitting - eyes closed - 120s
3. standing - eyes open - 3x (20s–20s break) - 45s break
4. standing - eyes closed - 3x (20s–20s break) - 45s break
5. standing, feet close together - 3x (20s–20s break) - 45s break
6. (semi-) tandem stand (feet behind each other) - 3x (20s–20s break) - 45s break
7. (semi) one-leg stand - 3x (20s–20s break) - 45s break break 5 min
8. 10 Perturbations Posturomed® - (30s–45s break) 2nd break 5 min.
9. 10 Perturbations Posturomed® - (30s–45s break)

Duration balance task: 12 min 30s; duration balance perturbation task: approx. 30 min.

The balance perturbation task will be performed on an oscillating sensorimotor therapy device (Bioswing Posturomed®, Haider, Pullenreuth, Germany) [20]. The task consists of reactive balance control after a previous balance perturbation (with manual triggering of the oscillating movements (see Table 6)). The oscillating mechanism enables dosed, damped pendulum movements with standardized oscillation amplitudes and frequencies (perturbations) [20]. The stabilization behavior is recorded by an acceleration sensor (3D Acceleration Sensor, Brain Products GmbH, Gilching, Germany). With regard to the attainment of postural stability, the postural corrective information from the start of movement of the platform, shall be calculated via the accelerometer data. During the balance perturbation task, perturbation-evoked cortical potentials will also be recorded via EEG.

In regard to technical specifications, the AccuGait-Optimized force plate (Advanced Mechanical Technology, Inc., Massachusetts, US), provides a measuring accuracy of .01 % according to the manufacturer, and is CE-certified [21]. Based on precision grid calibration technology, measurement accuracy is secured (10 separate force values for 400 locations on the platform surface) [21]. AMTI Balance Clinic measurement software will be used for data processing (Advanced Mechanical Technology, Inc., Massachusetts, US version, 1.5.0). Measures of CoP (average, range, standard deviation, path length, 95th percentile ellipse measure, velocity) will be calculated. The sampling rate will be 50 data sets per second, filtered with a fixed 100 Hz third-order analog filter. Three valid trials per condition will be collected, which is consistent with current methodological practice to maximize the reliability of CoP-based balance measures in neurological populations and minimize the risk for distort CoP outcomes due to fatigue [[22], [23], [24], [25]]. The number and duration of tasks and trials are specified in Table 6. The EEG system LiveAmp 32 and actiCAP slim/snap (Brain Products GmbH, Gilching, Germany) fulfills the requirements of Medical Device Directive 93/42/EEC (MDD), classification DIN EN 62304, class 1 [26]. Bioswing Posturomed® diagnostic device (Haider, Pullenreuth, Germany) is certified according to MDR 2017/745 class I medical device [20].

The secondary endpoints of the study will be the measurement of fatigue using the Fatigue Severity Scale (FSS) [27]. The FSS represents the gold standard for measuring fatigue [28]. In addition, the Multiple Sclerosis Quality of Life-54 Questionnaire is used as a disease-specific instrument (MSQoL-54), which supplements the SF-36 questionnaire for recording health-related quality of life with 18 MS-specific items [29]. The MSQoL-54 is a validated and established instrument in measuring quality of life in MS [29]. Pain will be measured using the visual analog scale (VAS) [30]. VAS is used because it represents a simple and frequently used method for measuring pain in clinical practice and is also regarded as a measure of the effectiveness of treatment and thus offers comparability to other studies [30]. Change in spasticity will be measured via Numeric Rating Scale (NRS) [31]. NRS is a validated and established instrument for the measurement of spasticity [31]. The self-assessment version of the ICF-based WHO Disability Assessment Schedule 2.0 (WHODAS 2.0) will be used to measure activity- and participation-related parameters [32]. WHODAS 2.0 is validated and links back to ICF domains [32]. In addition, an ICF-based EQUITEDO® assessment to be completed by the therapists as an external assessment will be used to document the process and results of the hippotherapy measures [33]. EQUITEDO® assessment is also validated and also linkable to ICF domains [33]. Furthermore, a self-constructed questionnaire for monitoring regularity, frequency, and content of the treatment as usual of both IG and CG across the full duration of the intervention (including follow-up) will be applied. Questionnaires will be filled out and processed digitally using two software programs Questback Unipark (Tivian XI Ltd., Cologne, Germany) and EQUITEDO® application (Research Institute for Inclusion through Physical Activity and Sports Ltd., Frechen, Germany).

### 2.6 Screening and randomization

Participant recruitment will take place using a flyer in local media, at the study sites and at the supporting partner organization, German Multiple Sclerosis Society (DMSG) (Hannover, Germany). Subsequently, interested candidates can contact the scientific study team via telephone or email. An initial telephone interview will be conducted using a standardized script, where eligibility is checked according to the inclusion and exclusion criteria. Inclusion and exclusion criteria are similar to the criteria in the MS-HIPPO study and can be seen in Table 3, Table 4 [14]. If an inclusion is possible, patients will subsequently be referred to a study site physician, who will carry out an initial examination and approve a medical clearance. The patients included, will be informed about the study protocol and asked for their written consent. Patients can withdraw from the study at any time; medically relevant withdrawals are forwarded to the monitor and responsible ethics committee. After initial contact, written informed consent and medical clearance of the participants, they will be randomly assigned to IG and CG. The allocation sequence will be generated by computer-generated random numbers. Individuals are randomized stratified by location, gender distribution of the disease and Expanded Disability Status Scale (EDSS) [34]. A code-generated random mechanism will be used for concealment. Scientific study personnel will generate the allocation sequence, enroll participants, and assign participants to interventions based on a stratified adaptive randomization.

### 2.7 Assessment

At baseline, post and follow-up, balance performance, BBS, EEG, quality of life, fatigue, pain, spasticity and activity- and participation-related parameters will be collected. The following table (Table 7) shows the time schedule of patient enrolment and intervention.

**Table 7:** Time schedule.

	Registration	Baseline assessment	Post assessment	Follow-up assessment
Fulfillment of the inclusion criteria	X	12 weeks hippotherapy or 12 weeks treatment as usual		6 weeks treatment as usual
Written declaration of informed consent	X			
Medical clearance	X			
Assignment to the study group	X			
Anthropometric data	X			
Balance assessment/EEG		X	X	X
BBS		X	X	X
Quality of life (MSQoL-54)		X	X	X
Fatigue (FSS)		X	X	X
Pain (VAS)		X	X	X
Spasticity (NRS)		X	X	X
WHODAS 2.0 36-Self		X	X	X
EQUITEDO® (IG only)		X	X	

### 2.8 Statistical procedures

The sample size was estimated on the basis of the primary outcome measure, the relevant effect size and the planned statistical test procedures. Statistical results of preliminary study MS-HIPPO showed a large effect in the per-protocol population with a change of 4.61 points in BBS between the IG and CG (95 % CI: .74–7.47, p = .002, N = 41) [11]. As individual-level baseline data were not available, a baseline–post correlation could not be calculated, and the sample size was therefore estimated based on the large empirical effect observed in the MS-HIPPO trial. Sample size was determined with G∗Power (Heinrich Heine University Duesseldorf, Germany, version 3.1.9.7) for the statistical F-test ANCOVA with two groups and five covariates, assuming an effect size of f = .40 (α = .05, 1–β = .80). This resulted in N = 111 (df = 104, actual power = .80), which was inflated to N = 140 to account for an anticipated 20% attrition rate, for the primary analysis model [35], consistent with methodological recommendations in clinical research using the standard factor ( *Nplanned* = 111 ÷ (1 − .20) = 139 ≈ 140), [11,36].

The study represents a confirmatory analysis, but due to the in-depth biomechanical and neurophysiological experimental measurements, the sample size calculation also includes a calculation of an additional sub-sample for the experimental protocol. In addition to the assessments listed in Table 7, they subsequently completed the balance tasks on the force plate and oscillating sensorimotor therapy device at each of the three measurement timepoints (see Table 6). In order to protect the patient collective of the study and avoid increased drop-out due to complex biomechanical and neurophysiological measurement technology, a reduced sample size was calculated on the basis of the effect strength of the preliminary study [11,14]. According to the effect size calculations of the Institute for Medical Statistics and Bioinformatics at the University of Cologne, the mean difference in BBS of 3.07 points (standard deviation (SD) = 1.04) of the IG and CG for a type 1 error rate of α = 1 % and a power of 1-β = 95 %, a required minimum sample size of N = 44 was calculated for the statistical F-test ANCOVA (fixed effects, main effects and interactions, two groups and five covariates) (95 % CI: 1.00–5.14, p = .004) assuming an effect size of f = 1.47 (df = 36, actual power = .95) [11,35]. Based on the ≈40 % drop-out rate of the preliminary study (N = 70) PP analysis (N = 41), a total of 70 patients (2x35) shall be randomized for in depth-biomechanical and neurophysiological measurements (during balance task and balance perturbation task) with a drop-out rate of around 40 %, to ensure a sufficient number of cases for this more extensive analysis [11]. Statistical evaluations will be carried out using the programs IBM SPSS 29 (IBM, Ehningen, Germany), R-Statistics (R Foundation, Vienna, Austria), JASP (JASP Team (2024), JASP (Version 0.19.3) [Computer software]) and Python [37]. Statistical methods for analyzing the primary outcome of change in balance performance after 12 units of therapy (change from baseline) will be covariance analyses (ANCOVA) (95 % confidence interval). The data will be analyzed with regard to an anchor-based approach of the minimal clinically important difference (MCID) of the BBS in individuals with MS (3 points according to Gervasoni et al., [38]. These analyses shall be verified by sensitivity analyses using linear mixed models (LMMs). Via this approach, incorporate fixed and random effects are being calculated to accurately represent non-independent data structures, to capture the change over the duration of therapy and follow-up. Therapeutic facility, gender, age, baseline BBS and EDSS score are included in the calculations as co-variables. This approach accommodates missing data under the missing at random (MAR) assumption, ensuring that all available measurements are included and therefore provide less biased and more robust estimates.

The secondary study endpoints will be examined using descriptive statistical methods. Furthermore, to complement the primary analysis, an analogous ANCOVA will also be performed for secondary study endpoints. For the quality of life questionnaire (MSQoL-54), an MCID of 1.5 and 2.5 points will be applied [39]. Three procedures are applied to analyze the study results: (1) the *modified* intention-to-treat population (*m*ITT), which comprises all registered patients who completed the pre-measurement and serves as the primary analysis set for this investigation. It represents a solution for managing complications as noncompliance or missing outcomes and therefore reflects the practical clinical scenario [40]. (2) The *per protocol* population (PP): This data set contains all patients who completed the study without or with very minor violations of the regulations according to the study program and is considered a sensitivity analysis. It is considered a protocol violation if less than 9 units of hippotherapy were completed. Analyses for primary and secondary endpoints will be conducted for *m*ITT and PP populations, whereas evaluations based on the PP-population will serve as sensitivity analyses. (3) The third data set includes the safety population and considers AEs; it includes patients who completed at least one treatment, as opposed to the treatment they were randomized to receive. Safety data (AEs) are summarized and documented according to type, severity, intensity and correlation [41].

### 2.9 Ethical aspects and monitoring

The trial will be conducted by researchers of the German Sport University (Cologne, Germany) together with Kamillus Hospital (Asbach, Germany). Study monitoring will be realized by a scientific partner institution (Medical School Berlin Germany(MSB)). It will ensure study quality and compliance with good clinical practice standards (GCP). Furthermore, the study will be designed and conducted in accordance with the German General Data Protection Regulation (GDPR) by the European Union. Unforeseen or adverse events in the therapy process will be documented by the therapists using the German version of the Serious Adverse Event (SAE) Report Form and forwarded to the study team within 24 h [41]. Important protocol amendments will be forwarded to the monitor, ethical committees and if relevant, to the participants directly after decision. Data will be stored on protected servers of the German Sports University Cologne in Germany. Data will be evaluated in a pseudonymized form by assigning a study number to each study participant. A separate list in which the study numbers are assigned to the participant data (name and contact details), as well as the declarations of consent, are only accessible to the leading hippotherapists at the study sites, e.g. to contact the patients as part of the treatment or in the event of revocation during the intervention time. For data analysis, only datasets that are coded with the study number will be processed. The assignment lists will be destroyed after the data collection has been completed and the research data will be destroyed after the usual retention period of 10 years. The included participants have the right to information (including the right to a free copy of the data), as well as correction and restriction of data processing in accordance with the GDPR.

## 3 Discussion

The proposed MS-HIPPO II study builds on the MS-HIPPO study, the first randomized controlled study to assess the effects of hippotherapy in people with MS on evidence level 1b [11,14]. The aim is to contribute to the current evidence by reliably, objectively and validly recording the effects of hippotherapy on balance performance based on a larger number of participants. Biomechanical and neurophysiological measurements should gain further knowledge of balance performance of persons with MS and allow precise assessment of potential change. The RCT follow-up study design shall contribute to the detection of permanence of potential effects. The collection of AEs should be providing more transparency about the course of therapy and its actual benefits. In the future, this study approach should contribute to further knowledge about hippotherapy as a non-pharmacological movement therapy approach for treating MS in outpatient rehabilitation. To optimize MS specific health care beyond pharmacological therapy, movement and physical activity related forms of therapy can potentially provide a valuable benefit in an integrated medical approach.

## CRediT authorship contribution statement

**Isabel Stolz:** Writing – original draft, Project administration, Methodology, Investigation, Funding acquisition, Conceptualization. **Leonard Braunsmann:** Writing – review & editing, Methodology, Investigation, Conceptualization. **Franca Rosiny:** Writing – review & editing, Software, Project administration, Investigation, Conceptualization. **Dieter Poehlau:** Writing – review & editing, Supervision, Conceptualization. **Thomas Abel:** Writing – review & editing, Supervision, Conceptualization. **Volker Anneken:** Writing – review & editing, Conceptualization. **Marion Drache:** Writing – review & editing, Conceptualization. **Kristel Knaepen:** Writing – review & editing, Methodology, Funding acquisition, Conceptualization.

## Ethics approval and consent to participate

The study was approved by ethics committee of the German Sport University Cologne (approval code 163/2023), and ethics commission Rhineland-Palatinate (approval code 2023–17325). Written informed consent to participate will be obtained from all participants. Written medical clearance will also be conducted by study physicians.

## Consent for publication

Written informed consent for publication will be obtained from all participants. A model consent form can be provided on request.

## Funding

The study is funded by Willi Drache Foundation, Johannisberg 1, 53578 Windhagen, Germany, Gold-Kraemer-Foundation, Paul-R.-Kraemer-Allee 100, 50226 Frechen, Germany, the German Curatorship for Therapeutic Riding (DKThR), Freiherr-von-Langen-Straße 8, 48231 Warendorf, Germany and the 10.13039/501100007458German Multiple Sclerosis Society (DMSG), Krausenstr. 50, 30171 Hannover, Germany.

## Declaration of competing interest

The authors declare the following financial interests/personal relationships which may be considered as potential competing interests:Investigators have no competing interest. Marion Drache is Chairwoman of Willi Drache Foundation. Dr. Volker Anneken is Managing Director of the Gold-Kraemer-Foundation. Both will not be part of the study investigators’ team.

## Figures and Tables

**Table A1:** Intervention manual for hippotherapy (DKThR)®

Domain	Specification
Dose	30 min mounted time per session; 1 session/week for 12 weeks (total = 12 sessions). Planned weekly progression: individualized adjustment of tasks/positions while maintaining session length.
Horse characteristics	The therapy horse must be specifically trained for hippotherapy, and be in good health with a well-muscled back. Its gait should be rhythmic, supple, and characterized by a steady, active walk. Horse size appropriate to rider's body height and weight and suitable for the ability to secure the rider on the horse. Horse stride frequency, overtrack and ground cover appropriate to rider's ability to adapt to the horse's movement. Predominant gait: walk, with even, rhythmic, three-dimensional movement. Temperament: calm, reliable, accustomed to therapeutic setting.
Tack & assistive devices	DKThR-approved therapy pad with handle; stirrups generally not used; use of belts, wedges, pads if required for positioning or safety. Helmets mandatory; the horse is guided on long reins.
Sequence of activities/positions/progression	Initial mounting with support; standard sequence includes: warm-up (rhythmic walk, symmetrical sitting), therapeutic tasks (balance exercises, trunk activation, reaching, transitional postures such as side-sitting or half-kneel as indicated), cool-down (relaxation in symmetrical seat). Progression criteria: ability to maintain trunk stability and execute tasks safely; increased complexity of tasks (e.g., reaching further, dynamic balance tasks) across weeks.
Team training & certification	All sessions delivered by DKThR-certified physiotherapists with additional training in hippotherapy; therapy horses trained and certified according to DKThR standards; equine assistants/side walkers instructed per site protocol. Facility certified by the DKThR. Team trained by scientific study team for study-related procedures and documenting with EQUITEDO® assessment-tool, AE and SAE report forms.
Per-session fidelity checklist	Therapy documented with EQUITEDO® assessment-tool, (Tack, tasks performed, patient response, adverse events). Fidelity monitored by site coordinator.
Monitoring of co-interventions	All concomitant physiotherapy is recorded at baseline, posttest and follow-up. Any change in medication or health-related treatments must be reported and can lead to exclusion.

## Data Availability

Investigators and monitor will have access to the final trial dataset. Restrictions apply to the availability of these data, which were used under license for the current study, and so are not publicly available.

## References

[1] Motl R.W. (2010). Physical activity and irreversible disability in multiple sclerosis. Exerc. Sport Sci. Rev..

[2] White L.J., Castellano V. (2008). Exercise and brain health--implications for multiple sclerosis: part 1--neuronal growth factors. Sports Med..

[3] Motl R.W., Sandroff B.M., Kwakkel G., Dalgas U., Feinstein A., Heesen C., Feys P., Thompson A.J. (2017). Exercise in patients with multiple sclerosis. Lancet Neurol..

[4] Pedersen B.K., Saltin B. (2015). Exercise as medicine - evidence for prescribing exercise as therapy in 26 different chronic diseases. Scand. J. Med. Sci. Sports.

[5] Pedersen B.K., Saltin B. (2006). Evidence for prescribing exercise as therapy in chronic disease. Scand. J. Med. Sci. Sports.

[6] Dalgas U., Langeskov-Christensen M., Stenager E., Riemenschneider M., Hvid L.G. (2019). Exercise as medicine in multiple sclerosis-time for a paradigm shift: preventive, symptomatic, and disease-modifying aspects and perspectives. Curr. Neurol. Neurosci. Rep..

[7] Learmonth Y.C., Motl R.W. (2021). Exercise training for multiple sclerosis: a narrative review of history, benefits, safety, guidelines, and promotion. Int. J. Environ. Res. Publ. Health.

[8] German Curatorship for Therapeutic Riding (2024). Description of the treatment measure hippotherapy. https://www.dkthr.de/produkt/hippotherapie-dkthr-2/.

[9] Federal Joint Committee (2006). Hippotherapy summary documentation on the evaluation of hippotherapy as a therapeutic appliance by the "Therapeutic appliances and aids" subcommittee of the joint federal committee. https://www.g-ba.de/downloads/40-268-126/2006-11-13-Abschluss-Hippo.pdf.

[10] Moraes A.G., Neri S.G.R., Motl R.W., Tauil C.B., von Glehn F., Corrêa É.C., de David A.C. (2021). Effects of hippotherapy on postural balance, functional mobility, self-perceived fatigue, and quality of life in people with relapsing-remitting multiple sclerosis: secondary results of an exploratory clinical trial. Mult. Scler. Relat. Disord..

[11] Vermöhlen V., Schiller P., Schickendantz S., Drache M., Hussack S., Gerber-Grote A., Pöhlau D. (2018). Hippotherapy for patients with multiple sclerosis: a multicenter randomized controlled trial (MS-HIPPO). Mult. Scler..

[12] Moutaftsis K., Trevlaki E., Chalkia A., Chandolias K., Trevlakis E., Leptourgos G., Papazoglou N. (2021). Hippotherapy in multiple sclerosis: a review focusing on motor function. Int. J. Sci. Res. Arch..

[13] Suárez-Iglesias D., Bidaurrazaga-Letona I., Sanchez-Lastra M.A., Gil S.M., Ayán C. (2021). Effectiveness of equine-assisted therapies for improving health outcomes in people with multiple sclerosis: a systematic review and meta-analysis. Mult. Scler. Relat. Disord..

[14] Wollenweber V., Drache M., Schickendantz S., Gerber-Grote A., Schiller P., Pöhlau D. (2016). Study of the effectiveness of hippotherapy on the symptoms of multiple sclerosis - outline of a randomised controlled multicenter study (MS-HIPPO). Contemp. Clin. Trials Commun..

[15] German Curatorship of Therapeutic Riding (2025). Implementation regulations (guidelines) of the German curatorship for therapeutic riding e.V. (DKThR) for equine-assisted therapy, support and Equestrian sport for persons with disabilities. https://dkthr.de/wp-content/uploads/2024/02/Durchfuehrungsbestimmungen_des_DKThR_Stand_Februar_2024.pdf.

[16] German Curatorship of Therapeutic Riding (2025). Certified institutions of the DKThR. https://dkthr.de/fachkraefte-und-anerkannte-einrichtungen-dkthr/anerkannte-einrichtungen-dkthr/.

[17] (2025). German curatorship of therapeutic riding e.V., self-presentation. https://dkthr.de/deutsches-kuratorium-fuer-therapeutisches-reiten/.

[18] Berg K., Wood-Dauphinee S., Williams J.I. (1995). The balance scale: reliability assessment with elderly residents and patients with an acute stroke. Scand. J. Rehabil. Med..

[19] Schaedler S. (2007). Assessment: berg balance scale. A revealing test for balance. Physiopraxis.

[20] Mueller O., Guenther M., Krauss I., Horstmann T. (2004). Physical characterization of the posturomed therapy device as a measuring instrument--presentation of a method for quantifying balance capacity. Biomed. Eng..

[21] Advanced Mechanical Technology, Inc. (2013). It Also Features a Clean and Simple USB Interface, Requiring Only One 15 Ft Cable for Both Data Transfer and Power.

[22] Gray V.L., Ivanova T.D., Garland S.J. (2014). Reliability of center of pressure measures within and between sessions in individuals post-stroke and healthy controls. Gait Posture.

[23] Sedaghati P., Alghosi M., Hosseini F. (2023). The effect of fatigue on postural control in individuals with multiple sclerosis: a systematic review. BMC Neurol..

[24] van Criekinge T., Heremans C., Burridge J., Deutsch J.E., Hammerbeck U., Hollands K., Karthikbabu S., Mehrholz J., Moore J.L., Salbach N.M., Schröder J., Veerbeek J.M., Weerdesteyn V., Borschmann K., Churilov L., Verheyden G., Kwakkel G. (2024). Standardized measurement of balance and mobility post-stroke: consensus-Based core recommendations from the third stroke recovery and rehabilitation roundtable. Int. J. Stroke.

[25] Ruhe A., Fejer R., Walker B. (2010). The test-retest reliability of centre of pressure measures in bipedal static task conditions--a systematic review of the literature. Gait Posture.

[26] Products Brain Operating instructions LiveAmp 32 and actiCAP slim/snap. https://www.brainproducts.com/downloads/manuals/.

[27] Krupp L.B., LaRocca N.G., Muir-Nash J. (1989). The fatigue severity scale. Application to patients with multiple sclerosis and systemic lupus erythematosus. Arch. Neurol..

[28] Pfeffer A. (2010). Assessment: fatigue severity scale – use for exhaustion. Ergopraxis.

[29] Vickrey B.G., Hays R.D., Harooni R., Myers L.W., Ellison G.W. (1995). A health-related quality of life measure for multiple sclerosis. Qual. Life Res..

[30] Carlsson A.M. (1983). Assessment of chronic pain. I. Aspects of the reliability and validity of the visual analogue scale. Pain.

[31] Bhimani R.H., Anderson L.C., Henly S.J., Stoddard S.A. (2011). Clinical measurement of limb spasticity in adults: state of the science. J. Neurosci. Nurs..

[32] Pösl M., Cieza A., Stucki G. (2007). Psychometric properties of the WHODASII in rehabilitation patients. Qual. Life Res..

[33] Stolz I., Anneken V., Froböse I. (2022). Measuring equine-assisted therapy: validation and confirmatory factor analysis of an ICF-based standardized assessment-tool. Int. J. Environ. Res. Publ. Health.

[34] Kurtzke J.F. (1983). Rating neurologic impairment in multiple sclerosis: an expanded disability status scale (EDSS). Neurology.

[35] Faul F., Erdfelder E., Buchner A., Lang A.-G. (2009). Statistical power analyses using G∗Power 3.1: tests for correlation and regression analyses. https://www.psychologie.hhu.de/arbeitsgruppen/allgemeine-psychologie-und-arbeitspsychologie/gpower.

[36] Bujang M.A. (2021). A step-by-step process on sample size determination for medical research. Malays. J. Med. Sci..

[37] Van Rossum G., Drake F.L. (1995).

[38] Gervasoni E., Jonsdottir J., Montesano A., Cattaneo D. (2017). Minimal clinically important difference of berg balance scale in people with multiple sclerosis. Arch. Phys. Med. Rehabil..

[39] Taheri M., Negahban H., Mostafaee N., Salehi R., Tabesh H. (2016). Responsiveness of selected outcome measures of participation restriction and quality of life in patients with multiple sclerosis. Disabil. Rehabil..

[40] Gupta S.K. (2011). Intention-to-treat concept: a review. Perspect. Clin. Res..

[41] Federal Institute for Drugs and Medical Devices, German SAE Report Form, 2018 ​https://www.bfarm.de/SharedDocs/Formulare/EN/MedicalDevices/report_form_clinical_trials_SAE.html.

